# Smartphone Screen Time Characteristics in People With Suicidal Thoughts: Retrospective Observational Data Analysis Study

**DOI:** 10.2196/57439

**Published:** 2024-10-11

**Authors:** Marta Karas, Debbie Huang, Zachary Clement, Alexander J Millner, Evan M Kleiman, Kate H Bentley, Kelly L Zuromski, Rebecca G Fortgang, Dylan DeMarco, Adam Haim, Abigail Donovan, Ralph J Buonopane, Suzanne A Bird, Jordan W Smoller, Matthew K Nock, Jukka-Pekka Onnela

**Affiliations:** 1 Department of Biostatistics, Harvard T.H. Chan School of Public Health, Harvard University, Boston, MA, United States; 2 Department of Psychology, Harvard University, Cambridge, MA, United States; 3 Department of Psychology, Rutgers University, Piscataway, NJ, United States; 4 Department of Psychiatry, Massachusetts General Hospital, Boston, MA, United States; 5 Department of Psychiatry, Harvard Medical School, Boston, MA, United States; 6 Center for Precision Psychiatry, Massachusetts General Hospital, Boston, MA, United States; 7 Franciscan Children's, Mental Health Research, Brighton, MA, United States; 8 National Institute of Mental Health, Bethesda, MD, United States; 9 Psychiatric and Neurodevelopmental Genetics Unit, Center for Genomic Medicine, Massachusetts General Hospital, Boston, MA, United States; 10 Stanley Center for Psychiatric Research, Broad Institute of MIT and Harvard, Boston, MA, United States

**Keywords:** smartphone, mobile apps, mobile health, screen time, suicidal thoughts and behavior, suicidal, app, observational data, data analysis study, monitor, survey, psychiatric, screen, mental health, feasibility, suicidal ideation, mobile phone

## Abstract

**Background:**

Smartphone-based monitoring in natural settings provides opportunities to monitor mental health behaviors, including suicidal thoughts and behaviors. To date, most suicidal thoughts and behaviors research using smartphones has primarily relied on collecting so-called “active” data, requiring participants to engage by completing surveys. Data collected passively from smartphone sensors and logs may offer an objectively measured representation of an individual’s behavior, including smartphone screen time.

**Objective:**

This study aims to present methods for identifying screen-on bouts and deriving screen time characteristics from passively collected smartphone state logs and to estimate daily smartphone screen time in people with suicidal thinking, providing a more reliable alternative to traditional self-report.

**Methods:**

Participants (N=126; median age 22, IQR 16-33 years) installed the Beiwe app (Harvard University) on their smartphones, which passively collected phone state logs for up to 6 months after discharge from an inpatient psychiatric unit (adolescents) or emergency department visit (adults). We derived daily screen time measures from these logs, including screen-on time, screen-on bout duration, screen-off bout duration, and screen-on bout count. We estimated the mean of these measures across age subgroups (adults and adolescents), phone operating systems (Android and iOS), and monitoring stages after the discharge (first 4 weeks vs subsequent weeks). We evaluated the sensitivity of daily screen time measures to changes in the parameters of the screen-on bout identification method. Additionally, we estimated the impact of a daylight time change on minute-level screen time using function-on-scalar generalized linear mixed-effects regression.

**Results:**

The median monitoring period was 169 (IQR 42‐169) days. For adolescents and adults, mean daily screen-on time was 254.6 (95% CI 231.4-277.7) and 271.0 (95% CI 252.2-289.8) minutes, mean daily screen-on bout duration was 4.233 (95% CI 3.565-4.902) and 4.998 (95% CI 4.455-5.541) minutes, mean daily screen-off bout duration was 25.90 (95% CI 20.09-31.71) and 26.90 (95% CI 22.18-31.66) minutes, and mean daily screen-on bout count (natural logarithm transformed) was 4.192 (95% CI 4.041-4.343) and 4.090 (95% CI 3.968-4.213), respectively; there were no significant differences between smartphone operating systems (all *P* values were >.05). The daily measures were not significantly different for the first 4 weeks compared to the fifth week onward (all *P* values were >.05), except average screen-on bout in adults (*P* value = .018). Our sensitivity analysis indicated that in the screen-on bout identification method, the cap on an individual screen-on bout duration has a substantial effect on the resulting daily screen time measures. We observed time windows with a statistically significant effect of daylight time change on screen-on time (based on 95% joint confidence intervals bands), plausibly attributable to sleep time adjustments related to clock changes.

**Conclusions:**

Passively collected phone logs offer an alternative to self-report measures for studying smartphone screen time characteristics in people with suicidal thinking. Our work demonstrates the feasibility of this approach, opening doors for further research on the associations between daily screen time, mental health, and other factors.

## Introduction

The widespread use of smartphones has created new opportunities for capturing social, behavioral, and cognitive phenotypes in free-living settings [1]. The ability to collect data from individuals in their natural environments, rather than in controlled laboratory settings, allows for a more complete ascertainment of an individual’s behavior. In addition, the use of smartphones allows for near-continuous data collection, enabling a more detailed and dynamic view of an individual’s behavior over time. This approach is well suited to study mental health disorders that are often characterized by a fluctuating and recurrent course [2].

Suicide is a complex mental health problem that is characterized as an “act of intentionally ending one’s own life” [3]. According to the National Institute of Mental Health, in 2021, more than 48,000 people died by suicide in the United States [4]. Suicide is often triggered by one or a combination of factors, including mental illness, substance use disorders, traumatic life events, and social isolation [3].

To date, in suicidal thoughts and behaviors (STB) research, smartphone-based monitoring has been primarily used to collect “active” data, which requires a participant to actively engage by contributing data entries via surveys. For example, smartphones can be used to collect ecological momentary assessment (EMA) data, which are frequent surveys on an individual’s thoughts, feelings, and behaviors in their natural environment [5,6]. EMA deployed through a smartphone app has been successfully used to understand potential risk factors for STB and characterize dynamics in suicidal ideation over time [7-13]. Importantly, the ability to deploy active data collection via smartphones and access EMA outcomes in real time has given rise to ethical and safety concerns, including when and how to intervene if a participant’s responses indicate an elevated risk during the course of the study [14].

The use of smartphone “passive” data, that is, data collected from smartphone sensors and logs without any active engagement from a participant, is less common in STB research. Existing studies have mostly relied on proprietary mobile apps and analytic methods [15]. Using smartphone log data to obtain detailed characteristics of an individual’s screen time is a potentially compelling application in STB studies due to the growing research focus on the association between screen time and mental health. Indeed, existing studies have demonstrated a link between increased levels of anxiety and depression and high smartphone screen time, measured either objectively or via a self-report [16,17]. In addition, objectively measured excessive screen time on mobile devices has been linked with a decreased quantity of sleep, which can further exacerbate mental health issues [18]. Interestingly, Rozgonjuk et al [19] reported that in a sample of 101 undergraduate university students, depression and anxiety symptom severity negatively correlated with the frequency of phone screen unlocking but were not related to total screen time; this finding suggests that not only screen-on total duration time but also screen-on use patterns are potentially relevant.

Data missingness is a common issue in digital health studies [20,21]. For smartphone passive data, Kiang et al [22] estimated missingness due to sensor noncollection rates to be 19% for accelerometer data and 27% for GPS data across 6 digital phenotyping studies. Sensor noncollection has been attributed to users’ behavior (eg, forgetting to charge a phone, disabling the GPS, and uninstalling the study app) and technological factors (the phone operating system restricting data collection). If the expected data volume or temporal coverage is fixed by study design (as was the case in these studies), one can identify smartphone sensor noncollection by comparing the expected versus observed data volume or temporal coverage. Determining missing data in the context of estimating smartphone screen time from phone state logs is more complicated, and without identifying the missingness, results may be biased. To the best of our knowledge, to date, no systematic approach has been proposed to determine the missingness status of smartphone state logs.

In this paper, we present methods for identifying and characterizing smartphone screen-on bouts, which we define as periods of consecutive smartphone use, in smartphone state logs. These methods were applied to a sample of adolescents (n=50) and adults (n=76) with suicidal thinking. Data were collected for up to 6 months from participants’ personal Android and iOS phones using the Beiwe smartphone app (Harvard University) [23]. Our methodological contributions are as follows. First, we present a method for preprocessing raw smartphone state logs into discrete screen-on bouts, capturing the duration and timing of each screen interaction, and handling missingness due to sensor non-collection; additionally, we performed multiple sensitivity analyses for preprocessing of raw logs. Second, we extracted several day-level metrics from the preprocessed bouts, including total screen time volume and fragmentation metrics that quantify the temporal distribution of screen use. Third, we compared screen time measures from early versus later stages of monitoring (which starts after the presentation of suicidal thinking) to identify use pattern shifts potentially associated with recent suicidal thoughts. Finally, we demonstrated how functional data analysis regression techniques can be used to analyze complex minute-level screen time outcomes. We estimated a time-varying effect of a daylight saving time (DST) change on the outcome using the natural experiment this event creates. The code for all preprocessing and analysis steps is openly available on GitHub [24].

## Methods

### Ethical Considerations

This study was conducted ethically and in accordance with relevant guidelines and regulations. Institutional review board approval from Harvard University (IRB18-1749) was obtained before the study began. All data collection and maintenance followed university, hospital, state, and national policies and regulations. Participants gave informed consent before taking part in any study procedures. All identifying information, such as names, initials, or hospital numbers, has been omitted from the data used in this analysis to protect participant privacy.

### Study Design and Population

The study recruited 2 samples of participants: adults from Massachusetts General Hospital’s Acute Psychiatry Service and children or adolescents (and their parents or guardians) from Franciscan Children’s psychiatric inpatient unit. Adult participants were eligible if they were 18 years or older, with other criteria including owning a smartphone and presenting to the emergency department with suicidal thoughts. Children and adolescents aged 12‐19 years were recruited from Franciscan Children’s, with eligibility criteria including smartphone ownership, parental consent for minors, child or adolescent assent, and presenting problems that included suicidal thoughts. The recruitment process involved initial identification and informed consent, and it was conducted via both an in-person manner and a remote manner (but fully remote during heightened periods of the COVID-19 pandemic). The recruitment process was designed to promote diversity among participants, without restrictions based on diagnosis or clinical history.

### Phone State Logs

Phone state logs were collected with the Beiwe smartphone app, the front end of the open-source Beiwe high-throughput digital phenotyping platform [23]. The Beiwe platform consists of smartphone apps for Android and iOS, a web-based platform for study administration, and a cloud-based back-end system for data storage and processing. Participants were assigned a Beiwe participant ID and had the Beiwe app installed during enrollment, which defined a study start date (Multimedia Appendix 1). The phone state logs were recorded with millisecond-level timestamps. iOS and Android phones report state logs differently (Table S1 in Multimedia Appendix 1). For Android, the logs capture “screen turned on” and “screen turned off” events; for iOS, the logs capture “locked” and unlocked” events. For iOS, the log also includes an event for each 1% change (positive or negative) in battery charge level.

### Screen Time Estimation

We define a “screen-on bout” for a smartphone as a period of consecutive screen use and a “screen-off bout” as a period of consecutive screen nonuse. Each screen-on bout is followed by a screen-off bout, and vice versa. To estimate the timing and duration of screen-on bouts for iOS, we used the time intervals between consecutive “unlocked” and “locked” event timestamps, while for Android, we used the time intervals between consecutive “screen turned on” and “screen turned off” event timestamps. During preprocessing, we imputed missing logs, removed bouts attributed to notification arrivals (for Android only), and capped screen-on bouts that lasted longer than 30 minutes (which corresponds to approximately the 97th percentile). A detailed description of our methods is provided in Multimedia Appendix 1. Our preprocessing steps closely resemble those previously presented by Kristensen et al [25]. For comparison, we also estimated timing and duration of screen-on bouts using three comparator approaches: (1) imputing missing logs and capping screen-on bout duration at 6 hours (instead of 30 minutes); (2) not imputing missing logs, that is, only considering consecutive pairs of matching events (“unlocked” and “locked” for iOS and “screen turned on” and “screen turned off” for Android), and not capping screen-on bout duration (ie, minimal preprocessing); and (3) not imputing missing logs with screen-on bout duration capped at 30 minutes.

### Daily Measures of Screen Time

To obtain daily measures of smartphone screen time, we used the estimated screen-on bouts and calculated 4 metrics: total screen-on time, average screen-on bout duration, average screen-off bout duration, and screen-on bout count. We defined a day as the period from midnight to midnight in the Coordinated Universal Time (UTC) time zone. The smartphone state logs were originally recorded in UTC, and we assumed the time zone choice would have no impact on the statistical analyses. The total screen-on time and screen-on bout count metrics capture the volume of screen time, while the average bout duration metrics reflect screen-on time accumulation patterns.

### Missing Data Labeling

A heuristic was developed to identify periods of missing phone state logs for iOS based on changes in battery charge level log events. Our approach assumes that the battery charge level is in one of four states: (1) decreasing, (2) increasing (when the phone is being charged), (3) constant at 0% (when the phone battery is fully depleted), and (4) constant at 100% (when the phone battery is fully charged and the charger is plugged in). First, we defined a minute of the monitoring time as “valid” based on either of two criteria: (1) phone’s battery level was changing at least 1% per hour (decreasing or increasing) or (2) the phone had been recently charged to 100% and not depleted battery for a period of at most 12 hours (likely having a charger plugged in). Minutes that did not meet these criteria were labeled as invalid. A “valid day” was defined as one with at least 1080 valid minutes (18 hours). Unlike for iOS, we were unable to define “valid minutes” for Android due to the absence of phone battery level change logs. Therefore, we only defined “valid days” for Android data as those consisting of at least 8 distinct hours that contained at least 1 screen-on bout. Only valid days were included in the statistical analyses.

### Statistical Data Analysis

The data analysis sample consisted of participants who provided at least 28 valid days of data. A day was considered valid if it belonged to a 28-day period with at least 14 valid days. We computed the number of days in the study observation period and the number of valid days of phone state logs for each participant and characterized these measures using the median and range, separately for adolescents, adults, and the combined sample.

To visualize daily measures over time, we aggregated the measures (median, 25th, 75th, 10th, and 90th percentile) across participants for each relative day since the discharge, separately for adolescents and adults. We used a 7-day moving average to smooth the sample statistics and improve readability.

To quantify population-level daily measures of screen time, we considered 4 different linear mixed-effects models (LMMs). Each model had a participant-specific daily measure as the outcome. The LMMs had different fixed-effect coefficients: model 1 had a fixed effect for an age group (adolescents, adults); model 2 had a fixed effect for an age group, operating system (Android, iOS), and their interaction; model 3 had a fixed effect for an age group, monitoring period (one of: the first four weeks after discharge period, from the fifth week up to 6 months period), and their interaction; and model 4 had a fixed effect for an age group, study monitoring period (one of: the first four weeks after discharge period, from the fifth week up to 6 months period), and their interaction. Each LMM included a participant-specific random intercept. Additionally, models 3 and 4 also had a subject-specific random slope for a study period. We fitted each of these 4 different LMMs separately for each of the 4 daily measures: total screen-on time, average screen-on bout duration, average screen-off bout duration, and screen-on bout count (natural logarithm-transformed), yielding a total of 4×4=16 model fits. Using these models, least squares means of daily measures were estimated across age groups, operating systems, and study periods of interest. We also quantified the contrasts of interest: the difference between age groups from model 1, the difference between operating systems across age groups from model 2, and the difference between (differently defined) study periods across age groups from models 3 and 4. The statistical significance of the contrasts of interest was evaluated using a significance level of α=.05.

We calculated screen-on time for each minute of each participant day using the timing and duration of screen-on bouts. The function-on-scalar generalized LMM (FoS-GLMM) was used to estimate the time-varying effect of daylight saving time (DST) on the probability of screen-on time at the minute level. We chose to study the effect of DST changes because they create a natural experiment that allows us to assess the feasibility of our method. Specifically, these changes provide a sudden and exogenous shift in the timing of daylight hours, which can impact individuals’ daily routines and phone use patterns. By examining the minute-level screen time outcome around these time changes, we explore whether our method captures changes in phone use.

In our FoS-GLMM, the outcome was a participant- and day-specific functional observation recorded on a minute-level discrete grid, with a value of 1 if any screen-on time was recorded for that minute and 0 otherwise. To quantify the effect of DST, we included a time-varying fixed-effect indicator for the change effect (1 if the functional observation was from a time period after the DST change and 0 otherwise) and a time-varying participant-specific intercept. The model was estimated separately at the start and end of DST (March and November of 2019‐2022, respectively) using data from ±14 days from the time change, and we performed a sensitivity analysis using data from ±7-day and ±28-day windows. We estimated FoS-GLMMs using the fast inference approach for longitudinal functional models proposed by Cui et al [26], and we made inferences about the time-varying fixed effect based on 95% joint confidence bands.

## Results

### Sample Characteristics

In total, 297 participants had the Beiwe app installed during enrollment. Of those, 247 participants had at least 1 valid day of data. The final analysis sample consisted of 126 participants (Table 1). The median age of the combined sample (adolescents and adults) was 22 (IQR 12-69) years, with adolescents having a median age of 15 (IQR 12-18) years and adults having a median age of 30 (IQR 18-69) years. The sample was predominantly female (n=81, 64.3%) and self-identified as White (n=101, 80.2%). Most participants (n=75, 59.5%) used smartphones with iOS, but the proportion of iOS users differed between the 2 subsamples, with 84% (42/50) in adolescents and 43.4% (33/76) in adults. The study observation period had a median duration of 169 (IQR 42-169) days, and participants had a median of 94 (IQR 29-167) days of valid smartphone state logs.

**Table 1.** Baseline demographic and observation period metadata characteristics of the analysis sample.^a^

Characteristic (statistic)	Statistic value		
	Adolescents (n=50)	Adults (n=76)	Combined (N=126)
Age (years), median (IQR)	15 (12-18)	30 (18-69)	22 (12-69)
**Sex, n (%)**			
Female (cisgender, she or her pron^b^ or unspecified)	35 (70)	40 (52.6)	75 (59.5)
Female (transgender)	0 (0)	3 (3.9)	3 (2.4)
Female (cisgender, other than she or her pron)	2 (4)	1 (1.3)	3 (2.4)
Male (cisgender, he or him pron or unspecified)	11 (22)	31 (40.8)	42 (33.3)
Male (transgender)	1 (2)	1 (1.3)	2 (1.6)
Male (cisgender, other than he or him pron)	1 (2)	0 (0)	1 (0.8)
**Self-identified race, n (%)**			
Asian	3 (6)	2 (2.6)	5 (4)
Black or African American	1 (2)	6 (7.9)	7 (5.6)
White	41 (82)	60 (78.9)	101 (80.2)
More than 1 race	1 (2)	2 (2.6)	3 (2.4)
Other	0 (0)	3 (3.9)	3 (2.4)
Unavailable	4 (8)	3 (3.9)	7 (5.6)
**Smartphone operating system, n (%)**			
iOS	42 (84)	33 (43.4)	75 (59.5)
Android	8 (16)	43 (56.6)	51 (40.5)
**Observation period metadata, median (IQR)**			
Days in observation period	169 (51-169)	169 (42-169)	169 (42-169)
Smartphone state logs (valid data days)	103 (34-158)	86 (29-167)	94 (29-167)

a The number of valid data days of smartphone state logs was determined differently for iOS and Android operating systems due to the differences in the type of smartphone state logs recorded.

b pron: gender pronouns use.

### Daily Measures of Screen Time

Table 2 summarizes the estimated population-level mean daily smartphone screen time. Adults had a mean total screen-on time of 271.0 minutes (4.52 hours), while adolescents had a mean 254.6 minutes (4.24 hours). Adults showed slightly higher average screen-on bout duration (mean 4.998 vs 4.233 minutes), slightly higher average screen-off bout duration (mean 26.90 vs 25.90 minutes), and slightly lower average screen-on bout count (natural logarithm transformed; mean 4.090 vs 4.192) compared to adolescents. None of these differences were statistically significant (Table S3 in Multimedia Appendix 1, rows 1‐4). Mean daily smartphone screen time measures across different phone operating systems and age groups (adults and adolescents) are shown in Table S2 in Multimedia Appendix 1 (rows 9‐24). There were no statistically significant differences in any of the 4 screen time measures across phone operating systems within any age group (Table S3 in Multimedia Appendix 1, rows 5‐12).

**Table 2.** Mean daily screen time measures separately for age groups (adolescents and adults).

Daily measure and age group		Daily measure mean (95% CI)
**Total screen-on time (minutes)**		
	Adolescent	254.6 (231.4-277.7)
	Adult	271.0 (252.2-289.8)
**Average screen-on bout (minutes)**		
	Adolescent	4.233 (3.565-4.902)
	Adult	4.998 (4.455-5.541)
**Average screen-off bout (minutes)**		
	Adolescent	25.90 (20.09-31.71)
	Adult	26.90 (22.18-31.62)
**Log**^a^ **(screen-on bout count)**		
	Adolescent	4.192 (4.041-4.343)
	Adult	4.090 (3.968-4.213)

a log(): natural logarithm transformation.

**Figure 1.**
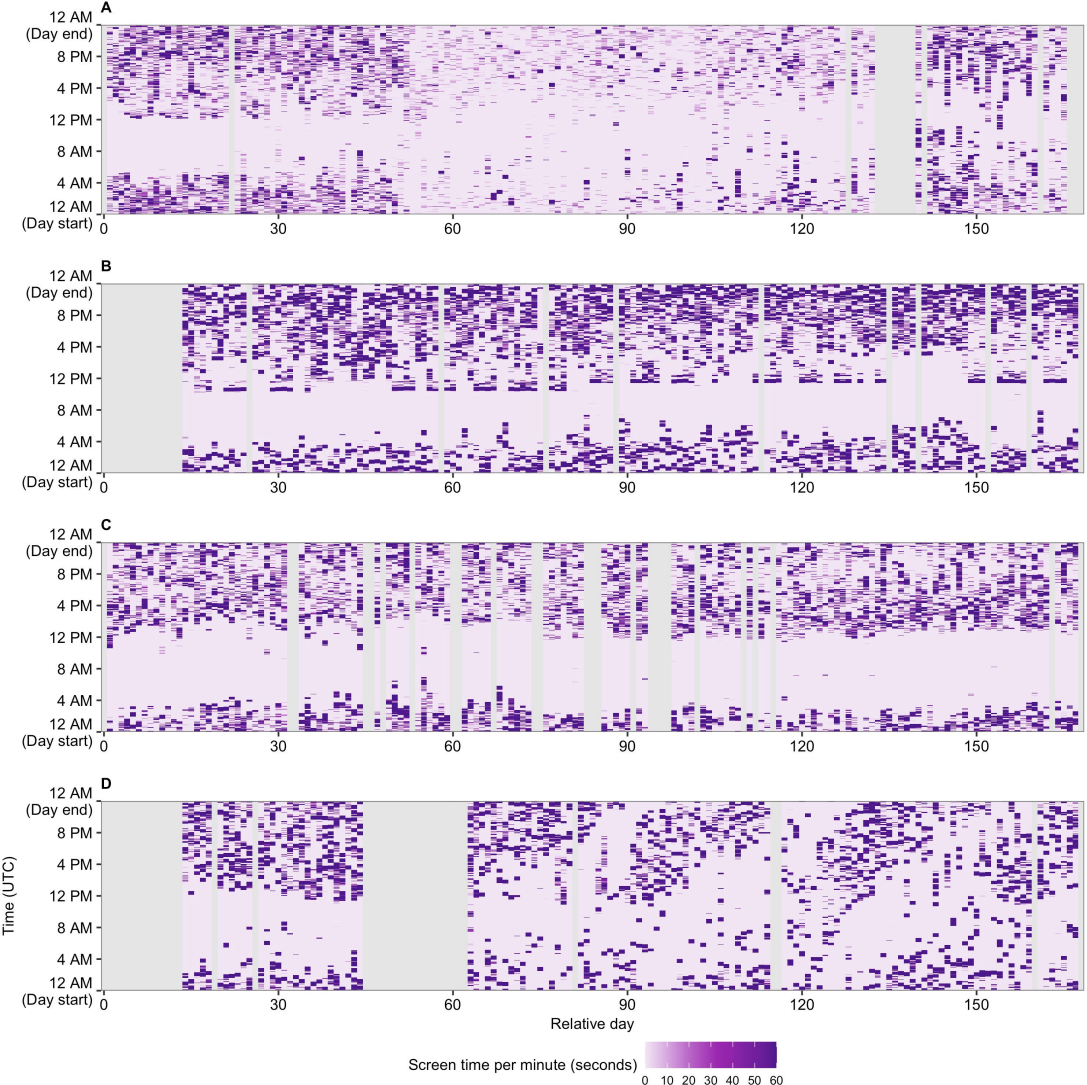
Characteristics of minute-level smartphone screen time for 4 participants. Screen time per minute is shown (color-coded; expressed in seconds) across minutes of the day (y-axis; UTC time) for each day of participant’s monitoring period relatively to the study start (x-axis). Days labeled as invalid data days are shadowed in gray. Plot A: ID 48 (iOS, adolescent) with the following means of daily measures: total screen-on time=166.7 minutes, average screen-on bout=0.9 minutes, and average screen-off bout=8.4 minutes. Plot B: ID 92 (iOS, adolescent): total screen-on time=351.0 minutes, average screen-on bout=4.3 minutes, and average screen-off bout=13.8 minutes. Plot C: ID 20 (iOS, adolescent): total screen-on time=253.6 minutes, average screen-on bout=2.5 minutes, and average screen-off bout=12.2 minutes. Plot D: ID 102 (iOS, adolescent): total screen-on time=266.7 minutes, average screen-on bout=8.8 minutes, and average screen-off bout=43.8 minutes. UTC: Coordinated Universal Time.

Figure 1 displays heat maps of minute-level smartphone screen time for 4 participants. Screen time per minute is shown across minutes of a day for each day of the participant’s observation period. The figure highlights various between- and within-participant screen time patterns. Participant ID 48 (plot A) has a relatively low average total screen-on time (mean 166.7 minutes across the monitoring days) and short average screen-on bouts (mean 0.9 minutes), whereas participant ID 92 (plot B) has a relatively high average total screen-on time (mean 351.0 minutes) and a moderate average screen-on bout duration (mean 4.3 minutes). Participant ID 20 and ID 102 (plots C and D) have similar total screen-on times (mean 253.6 and 266.7 minutes, respectively), but different accumulation patterns, as measured with average screen-on bout durations (mean 2.5 and 8.8 minutes, respectively) and average screen-off bout durations (mean 12.2 and 42.8 minutes, respectively). Participant ID 48 experiences a decline in daily total screen-on time around their 50th day relative to the study start. Participant ID 92 exhibits a pattern of waking up at the same time across many of the 5-day long periods of time (for 15th-80th relative days: waking up at 10:30 AM UTC, ie, 6:30 AM in their current Eastern Daylight Time [EDT] time zone; and for 80th-180th relative days: waking up at 11:30 AM UTC, ie, 6:30 AM in their current EST time zone). Participant ID 102 appears to have very irregular sleep and wake-up time and has screen-on time bouts scattered across the Eastern Time nighttime on multiple days.

**Figure 2.**
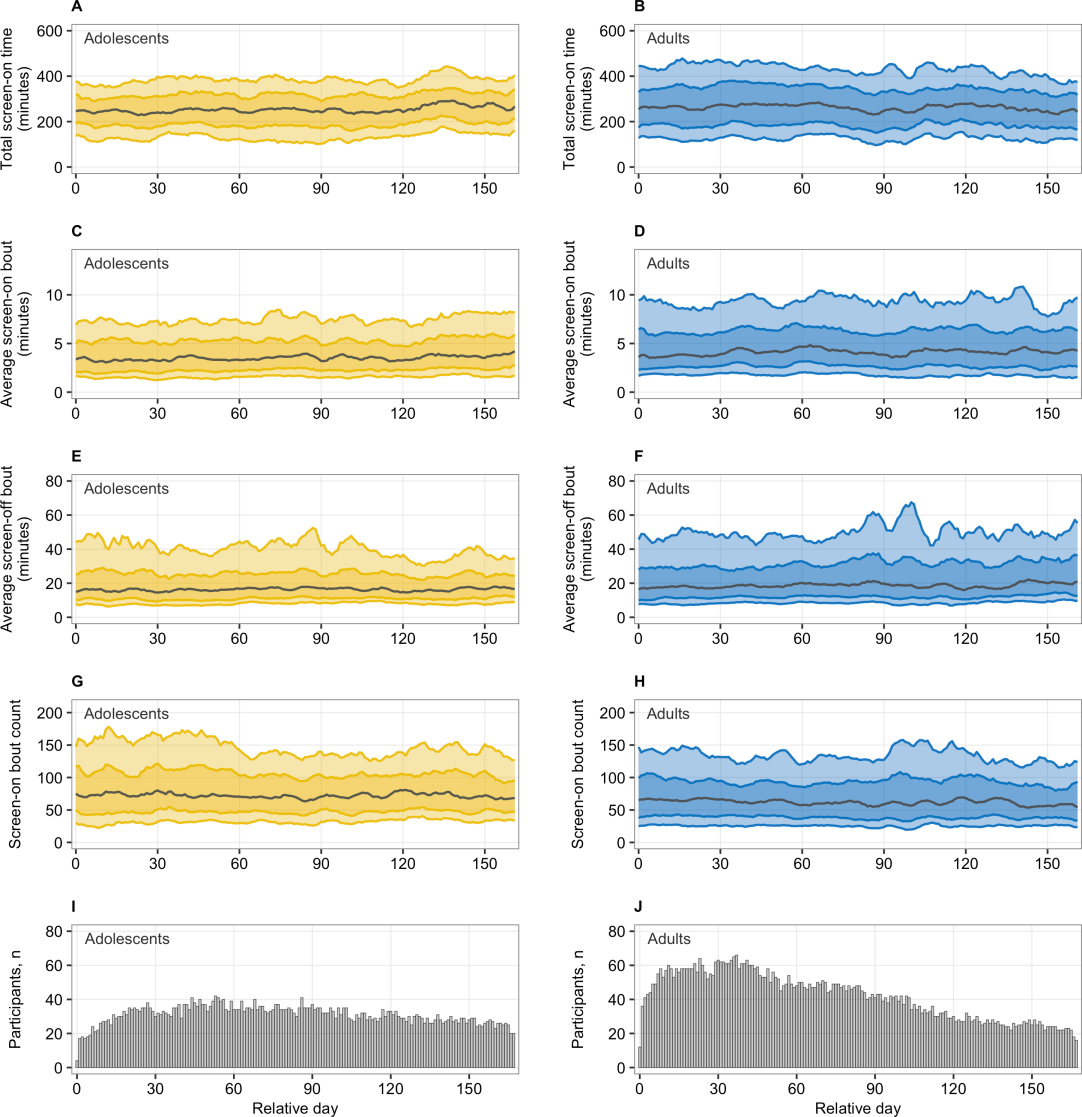
Characteristics of daily screen time measures across time relative from study start. Plots A-H show sample median (black line), 25th-75th percentile bounds (darker color ribbon), and 10th-90th percentile bounds (lighter color ribbon) of day-level measures: total screen-on time (in minutes; plots A and B), average screen-on bout duration (in minutes; plots C and D), average screen-off bout duration (in minutes; plots E and F), and screen-on bout count (plots G and H). Plots I and J show the number of participants contributing a valid day of phone state logs data on a given day relative from the study start.

Figure 2 displays the median, 25th-75th percentile bounds, and 10th-90th percentile bounds of the screen time daily measures aggregated across participants for each day relative to the study start, separately for adolescents and adults. Although some day-to-day variability is evident, we did not observe any specific patterns during the early versus later monitoring period, which is consistent with the results of our LMMs. Our analysis did not identify any statistically significant differences in population-level daily measures between the first 4 weeks and week 5 and onward nor between the first week and week 5 and onward after the discharge, except for 2 cases: in the adult subsample, the average screen-on bout duration was significantly lower during the first 4 weeks than week 5 and onward; and in the adolescent subsample, the average screen-off bout duration was significantly higher during the first 4 weeks than week 5 and onward. Please refer to Table S2 in Multimedia Appendix 1 for daily measure means across different treatment periods by age group (rows 25‐56) and to Table S3 in Multimedia Appendix 1 for difference between treatment periods by age group (rows 13‐28).

In addition to our proposed approach, we estimated the timing and duration of screen-on bouts using 3 comparator methods. Figure S1 in Multimedia Appendix 1 illustrates the differences in daily measures of total screen-on time and average screen-on bout duration among participants. Comparators 1 and 2 produced substantially higher estimates for daily total screen-on time, with mean differences of −106.1 (range −502.6 to −1.3) minutes and −133.5 (range −666.4 to −2.4) minutes, respectively. These methods used either a generous cap of 6 hours on an individual screen-on bout duration (comparator 1) or no cap at all (comparator 2). In contrast, comparator 3 (no imputation, capping screen-on bout duration at 30 minutes) yielded results similar to our proposed approach, with an average difference of −0.2 (range −11.8 to 7.3) minutes.

### Time-Varying Effect of a Daylight Time Change on Minute-Level Screen Time

**Figure 3.**
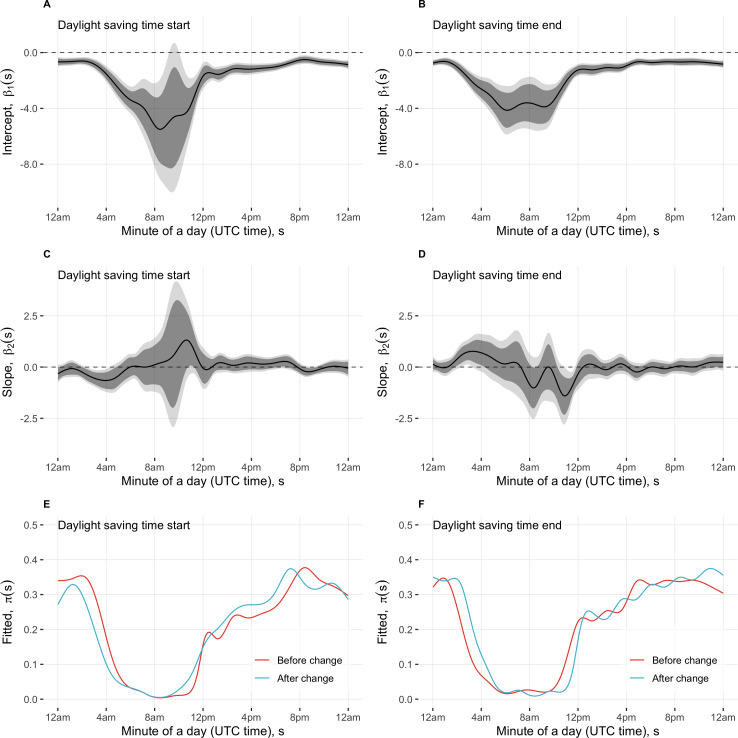
Estimates of functional intercept and slope for daylight saving time (plots A-D) and fitted probabilities of screen time (plots E and F) in a given minute across the minute-level functional domain of a day. Logistic function-on-scalar mixed-effects regression was used to estimate the time-varying effect of the covariate (1=after and 0=before daylight saving time change) on the functional outcome (1=any screen time and 0=no screen time). In rows 1‐2 (plots A-D), functional coefficient estimates (solid black line), 95% point-wise CIs (dark gray shaded area), and 95% joint CIs (light gray shaded area) are presented. In row 3 (plots E and F), fitted probabilities for before and after the change are shown, with vertical dashed lines indicating the time window with a statistically significant effect based on joint CIs. Separate models were fitted for the start (column 1; plots A, C, and E) and end (column 2; plots B, D, and F) of daylight saving time, each using a ±14-day window of data relative to the change night. UTC: Coordinated Universal Time.

We used FoS-GLMM to estimate a time-varying effect of a daylight time change on the probability of screen-on time at the minute level. Figure 3 shows estimates of a functional intercept and a functional slope for the fixed-effect covariate (1=after and 0=before DST change; plots A-D) as well as fitted probabilities of screen time (plots E and F) in a given minute across the functional domain of a day.

With the start of the DST model, we observed statistically significant negative effects (based on 95% joint confidence bands) on the probability of screen-on time after the time change during the period from 2:24 AM UTC to 4:19 AM UTC (equivalent to 9:24 PM EST to 11:19 PM EST before the time change). In this timeframe, the functional coefficient estimate had an average value −0.57 (range −0.65 to −0.38). This estimate suggests that the odds of phone use after the time change were 0.56 times lower compared to the odds before the time change. The average probabilities of phone use in a given minute were 0.26 and 0.17 for before and after the change in that period, respectively, as depicted by the blue and red lines in Figure 3E, respectively (located between the dashed vertical lines).

With the end of the DST model, we observed statistically significant positive effects (based on 95% joint confidence bands) on the probability of screen-on time after the time change from 2:22 AM UTC to 3:19 AM UTC (10:24 PM EDT to 11:19 PM EDT before the change) and statistically significant negative effects (based on 95% joint confidence bands) during the time window from 10:52 AM UTC to 11:44 AM UTC (6:52 AM EDT to 7:44 AM EDT before the change). In the time window of positive effect significance, the functional coefficient estimate had an average value 0.71 (range 0.58-0.77), corresponding to the odds of phone use after the time change being 2.03 times the odds before the change, and average probabilities of phone use in a given minute 0.15 and 0.27 for before and after the change, respectively (Figure 3E). In the time window of negative effect significance, the functional coefficient estimate had an average value −1.13 (range −1.40 to −0.66), corresponding to odds of phone use after the time change being 0.32 times the odds before the change, and average probabilities of phone use in a given minute 0.15 and 0.06 for before and after the change, respectively (Figure 3E).

Based on sensitivity analysis results (Figure S2 in Multimedia Appendix 1), the inference was very similar for cases of using ±14 days (main analysis) and ±28 days, whereas for the ±7-day model, the 95% joint confidence bands do overlap with 0 value for the functional coefficient in the DST end model.

Taken together, these results suggest that the effects on screen-on time during these time windows are plausibly attributable to sleep time adjustments related to clocks going 1 hour forward (“we sleep less”) or 1 hour backward (“we sleep more”).

## Discussion

### Screen Time Data in STB Studies

Digital phenotyping entails the collection and analysis of various types of data from personal digital devices in naturalistic settings; it has applications both within and outside mental health. The focus of our paper is the development of a simple method to enable investigators to use moment-by-moment screen time metrics when studying adolescents and adults with STB. We emphasize that in our approach, the data are collected in naturalistic settings and arise as a byproduct of how participants use their phones, which makes the approach scalable.

In the digital phenotyping literature in mental health, there is surprisingly little existing research on the intersection of STB and screen time. For example, a recent literature review only briefly mentions STB and does not discuss screen time [27]. Another paper focusing on stress, anxiety, and mild depression carried out a systematic review of 40 studies [28]; 7 studies with “student participants” and 2 studies with “adult participants” used screen time data (screen on or off, phone lock or unlock, and similar metrics), but none of the reviewed studies dealt with STB. Finally, a recent narrative review of digital phenotyping for differential diagnosis of major depressive disorder reviewed 74 papers dealing with “digital tools”; 4 of the papers focused on STB, but none were reported to discuss screen time [29].

### Principal Findings

We investigated the smartphone screen time characteristics in 126 adolescents and adults with suicidal thinking. Passively collected smartphone state logs data for a median of 169 (IQR 42‐169) days provided objective measurements of screen time. Our analysis showed that study participants spent an average of 254.6 and 271.0 minutes per day on their smartphones for adolescents and adults, respectively. The means of participants’ average screen-on bout duration were 4.2 and 5.0 minutes, average screen-off bout duration were 25.9 and 26.9 minutes, and screen-on bout count (natural logarithm transformed) were 4.2 and 4.1 for adolescents and adults, respectively.

The daily measures remained relatively constant across the monitoring period. The near constancy of the measures at the daily level could be perceived as a favorable result. First, nearly all methods that attempt to detect changes in temporal data, such as anomaly detection and change-point detection methods, need to establish an underlying trend of the measure over time. Substantial variation in the daily measures of screen time could complicate the detection of changes potentially due to STB episodes. With that, there were very few suicide attempts in this cohort during the monitoring period (data not reported in this paper), highlighting the clinical team’s effectiveness in ensuring participant safety. Finally, the crucial question of the longitudinal association between screen time and STB, as well as the identification of STB episodes, will be addressed in future work, with this paper laying the groundwork by introducing the necessary method.

Another finding was the significant impact of DST changes on screen use behavior. When DST started, a decrease in screen-on time probability was observed between 2:24 AM and 4:19 AM UTC, with average probabilities dropping from 0.26 to 0.17. Conversely, when DST ended, an increase in screen-on time probability occurred between 2:22 AM and 3:19 AM UTC, with average probabilities rising from 0.15 to 0.27. A further decrease was observed between 10:52 AM and 11:44 AM UTC, with average screen-on probabilities dropping from 0.15 to 0.06. These findings suggest that sleep adjustments related to DST shifts do influence screen use behaviors.

Our study highlights the advantages of phone log–derived measures of screen time compared to traditional self-report surveys. As noted by Harris et al [30-33], self-report scales often lack internal consistency and test-retest reliability, rely on the participant’s memory, and may result in both over- and underestimation of screen time. In contrast, phone logs provide an objectively measured representation of an individual’s smartphone screen time, making them particularly useful for identifying trends or patterns in screen time, especially in longitudinal studies and free-living settings.

Our framework derives 4 daily measures of screen time: total screen-on time, average screen-on bout duration, average screen-off bout duration, and screen-on bout count to enable us to characterize a phone use behavior. The framework allows adaptation for different time resolutions, allowing researchers to explore subday-level (including minute-level) use patterns. For instance, they can investigate diurnal variations in screen time, identify atypical use periods, or study the impact of specific events on phone interactions. In future work, we propose to use sudden deviations from a participant’s routine of phone use (and screen time in particular) to identify potential STB episodes, with the goal of ultimately developing clinical interventions.

Finally, to the best of our knowledge, this is the first presentation of freely available code for preprocessing and analysis of raw phone state logs [24]. Using open-source software and sharing publicly the analysis code are essential for ensuring transparency and replicability of the results, promoting accountability, and building trust in the research process.

### Limitations

The primary limitation is the lack of a gold standard for identifying missing smartphone state logs. Though related mental health research often fails to report data quality, accounting for missingness is crucial for accurate screen time estimates and valid comparisons [16]. We proposed a heuristic for iOS to identify missingness at the minute level based on battery level changes and a method for both platforms to label data quality by day. Additionally, we evaluated the sensitivity of screen time measures to missing log imputation and, inherently related, the choice of maximum screen-on bout duration threshold (Figure S1 in Multimedia Appendix 1). Results showed substantial variability in participants’ mean total screen-on time and screen-on bout duration based on the chosen screen-on duration cap. Given the lack of a principled approach for selecting an optimal cap, researchers should be mindful that this choice can substantially influence measure estimates. We do not have a definitive reason for choosing the 30-minute threshold instead of other options. However, we believe this choice strikes a good balance by indicating when a long screen time session has likely occurred while minimizing the impact of outliers on our daily estimates. Our code is openly available for transparency.

We acknowledge the potential for differences in daily measures resulting from difference in how we identified on-screen bouts for iOS versus Android devices. In additional analyses (Tables S2 and S3 in Multimedia Appendix 1), we quantified the differences in daily measures between Android versus iOS across age groups (adolescents and adults). However, the observed difference sizes were relatively small, and no statistical significance between iOSs was found. It is important to note that the absence of statistical significance does not necessarily imply the absence of a true effect. Nevertheless, given the substantial sample size, we deemed the results sufficient for our analysis.

In the sensitivity analysis for time-varying effect of a daylight time change on minute-level screen time (Figure S2 in Multimedia Appendix 1), for the ±7-day model, the 95% joint confidence bands do overlap with 0 value for the functional coefficient in the DST end model. We note that the ±7-day model’s insignificant results might be due to either small sample size or using conservative 95% joint confidence bands to make inferences about the subset of the whole functional domain. Future studies could consider joint confidence bands for a prespecified restricted subset of the functional domain to increase power.

### Conclusions

Passively collected smartphone logs allowed us to estimate daily measures of screen time characteristics in a large sample of adolescents and adults with suicidal thinking over a half year–long monitoring period. Our work demonstrates the feasibility of this approach, opening doors for further research on the associations between daily screen time, mental health, and other factors.

## Supplementary material

10.2196/57439 Multimedia Appendix 1 Supplementary material containing further data on smartphone state log characteristics, estimated daily measures, screen-on time and bouts, the effect of daylight saving time, and additional details regarding our methodology.

## Abbreviations

DST: daylight saving time
EDT: Eastern Daylight Time
EMA: ecological momentary assessment
FoS-GLMM: function-on-scalar generalized linear mixed-effects model
LMM: linear mixed-effects model
STB: suicidal thoughts and behaviors
UTC: Coordinated Universal Time
