# Supplementary material for: Smartphone Screen Time Characteristics in People With Suicidal Thoughts: Retrospective Observational Data Analysis Study
Source: JMIR Mhealth Uhealth. 2024 Oct 11;12:e57439. doi: 10.2196/57439 (PMC11488461; doi:10.2196/57439)
Supplement: Multimedia Appendix 1 [file mhealth-v12-e57439-s001.docx]

**Table S1**. Characteristics of smartphone state logs across phone operating systems (OS). For Android OS, the "Value" column represents the value of a public static final string field of the android.content.Intent class in Java. For iOS OS, the "Value" column represents an enum value of UIDevice.BatteryState in Swift. The "Beiwe log statement label" table column shows the text representing the log in the raw data retrieved from the Beiwe platform, and the "Battery level" column indicates whether the current battery charge level is available in a given log.

| **Phone OS** | **Value** | **Beiwe log statement label** | **Battery level** |
| --- | --- | --- | --- |
| Android | ACTION_SCREEN_ON | Screen turned on | Not available |
| Android | ACTION_SCREEN_OFF | Screen turned off | Not available |
| Android | ACTION_POWER_DISCONNECTED | Power disconnected | Not available |
| Android | ACTION_POWER_CONNECTED | Power connected | Not available |
| Android | ACTION_POWER_SAVE_MODE_CHANGED (Android version 5.0+; in phone not in power save state) | Power Save Mode change signal received; device not in power save state. | Not available |
| Android | ACTION_POWER_SAVE_MODE_CHANGED (Android version 5.0+; in phone in power save state) | Power Save Mode state change signal received; device in power save state. | Not available |
| Android | ACTION_DEVICE_IDLE_MODE_CHANGED (Android version 6.0+; in phone not in idle state) | Device Idle (Doze) state change signal received; device not in idle state. | Not available |
| Android | ACTION_DEVICE_IDLE_MODE_CHANGED (Android version 6.0+; in phone in idle state) | Device Idle (Doze) state change signal received; device in idle state. | Not available |
| Android | ACTION_REBOOT | Device rebot signal received |  |
| Android | ACTION_SHUTDOWN | Device shut down signal received | Not available |
| iOS |  | Unplugged | Available |
| iOS |  | Charging | Available |
| iOS |  | Locked | Available |
| iOS |  | Unlocked | Available |
| iOS |  | Full | Available |

**Table S2**. Estimated daily measure means across different sample strata by age group (adolescent vs. adult), operating system (iOS vs. Android), study monitoring period (Weeks 1-4 vs. Weeks >=5, and Week 1 vs. Weeks >=5). Means were estimated for the following daily screen time measures: total screen-on time (minutes), average screen-on bout duration (minutes), average screen-off bout duration (minutes), screen-on bout count (natural logarithm-transformed). The model fit number ("Model fit no.") represents the index of an individual linear mixed-effects model fit used to estimate mean and 95% confidence interval of the mean in a given row.

| Row  no. | Model fit no. | Daily measure  (model outcome) | Age group (model covariate) | Operating system (model covariate) | Study period (model covariate) | Daily measure mean [95% CI]  (model estimate) |
| --- | --- | --- | --- | --- | --- | --- |
| 1 | 1 | Total screen-on time [min.] | Adolescent |  |  | 254.6 [231.4, 277.7] |
| 2 | 1 | Total screen-on time [min.] | Adult |  |  | 271.0 [252.2, 289.8] |
| 3 | 2 | Avg. screen-on bout [min.] | Adolescent |  |  | 4.233 [3.565, 4.902] |
| 4 | 2 | Avg. screen-on bout [min.] | Adult |  |  | 4.998 [4.455, 5.541] |
| 5 | 3 | Avg. screen-off bout [min.] | Adolescent |  |  | 25.90 [20.09, 31.71] |
| 6 | 3 | Avg. screen-off bout [min.] | Adult |  |  | 26.90 [22.18, 31.62] |
| 7 | 4 | Log(screen-on bout count) | Adolescent |  |  | 4.192 [4.041, 4.343] |
| 8 | 4 | Log(screen-on bout count) | Adult |  |  | 4.090 [3.968, 4.213] |
| 9 | 5 | Total screen-on time [min.] | Adolescent | iOS |  | 255.5 [230.1, 281.0] |
| 10 | 5 | Total screen-on time [min.] | Adolescent | Android |  | 249.6 [191.3, 307.9] |
| 11 | 5 | Total screen-on time [min.] | Adult | iOS |  | 267.7 [238.9, 296.5] |
| 12 | 5 | Total screen-on time [min.] | Adult | Android |  | 273.6 [248.4, 298.8] |
| 13 | 6 | Avg. screen-on bout [min.] | Adolescent | iOS |  | 4.258 [3.523, 4.994] |
| 14 | 6 | Avg. screen-on bout [min.] | Adolescent | Android |  | 4.101 [2.417, 5.786] |
| 15 | 6 | Avg. screen-on bout [min.] | Adult | iOS |  | 4.918 [4.087, 5.748] |
| 16 | 6 | Avg. screen-on bout [min.] | Adult | Android |  | 5.059 [4.332, 5.787] |
| 17 | 7 | Avg. screen-off bout [min.] | Adolescent | iOS |  | 26.92 [20.55, 33.29] |
| 18 | 7 | Avg. screen-off bout [min.] | Adolescent | Android |  | 20.55 [5.958, 35.14] |
| 19 | 7 | Avg. screen-off bout [min.] | Adult | iOS |  | 26.43 [19.23, 33.64] |
| 20 | 7 | Avg. screen-off bout [min.] | Adult | Android |  | 27.26 [20.95, 33.57] |
| 21 | 8 | Log(screen-on bout count) | Adolescent | iOS |  | 4.175 [4.009, 4.340] |
| 22 | 8 | Log(screen-on bout count) | Adolescent | Android |  | 4.281 [3.901, 4.661] |
| 23 | 8 | Log(screen-on bout count) | Adult | iOS |  | 4.130 [3.942, 4.317] |
| 24 | 8 | Log(screen-on bout count) | Adult | Android |  | 4.060 [3.896, 4.224] |
| 25 | 9 | Total screen-on time [min.] | Adolescent |  | Weeks 1-4 | 245.3 [219.6, 271.1] |
| 26 | 9 | Total screen-on time [min.] | Adolescent |  | Weeks >=5 | 256.7 [232.8, 280.6] |
| 27 | 9 | Total screen-on time [min.] | Adult |  | Weeks 1-4 | 265.4 [244.9, 285.9] |
| 28 | 9 | Total screen-on time [min.] | Adult |  | Weeks >=5 | 272.3 [252.8, 291.8] |
| 29 | 10 | Avg. screen-on bout [min.] | Adolescent |  | Weeks 1-4 | 4.034 [3.336, 4.731] |
| 30 | 10 | Avg. screen-on bout [min.] | Adolescent |  | Weeks >=5 | 4.234 [3.551, 4.918] |
| 31 | 10 | Avg. screen-on bout [min.] | Adult |  | Weeks 1-4 | 4.702 [4.145, 5.259] |
| 32 | 10 | Avg. screen-on bout [min.] | Adult |  | Weeks >=5 | 5.074 [4.518, 5.630] |
| 33 | 11 | Avg. screen-off bout [min.] | Adolescent |  | Weeks 1-4 | 30.86 [23.02, 38.70] |
| 34 | 11 | Avg. screen-off bout [min.] | Adolescent |  | Weeks >=5 | 24.74 [18.80, 30.68] |
| 35 | 11 | Avg. screen-off bout [min.] | Adult |  | Weeks 1-4 | 25.75 [19.56, 31.94] |
| 36 | 11 | Avg. screen-off bout [min.] | Adult |  | Weeks >=5 | 27.40 [22.55, 32.24] |
| 37 | 12 | Log(screen-on bout count) | Adolescent |  | Weeks 1-4 | 4.182 [4.014, 4.351] |
| 38 | 12 | Log(screen-on bout count) | Adolescent |  | Weeks >=5 | 4.202 [4.049, 4.354] |
| 39 | 12 | Log(screen-on bout count) | Adult |  | Weeks 1-4 | 4.120 [3.985, 4.255] |
| 40 | 12 | Log(screen-on bout count) | Adult |  | Weeks >=5 | 4.079 [3.955, 4.203] |
| 41 | 13 | Total screen-on time [min.] | Adolescent |  | Week 1 | 257.8 [226.6, 289.0] |
| 42 | 13 | Total screen-on time [min.] | Adolescent |  | Weeks >=5 | 256.8 [232.8, 280.7] |
| 43 | 13 | Total screen-on time [min.] | Adult |  | Week 1 | 258.4 [235.8, 281.0] |
| 44 | 13 | Total screen-on time [min.] | Adult |  | Weeks >=5 | 272.5 [253.0, 292.1] |
| 45 | 14 | Avg. screen-on bout [min.] | Adolescent |  | Week 1 | 4.367 [3.440, 5.294] |
| 46 | 14 | Avg. screen-on bout [min.] | Adolescent |  | Weeks >=5 | 4.227 [3.544, 4.911] |
| 47 | 14 | Avg. screen-on bout [min.] | Adult |  | Week 1 | 4.642 [3.963, 5.320] |
| 48 | 14 | Avg. screen-on bout [min.] | Adult |  | Weeks >=5 | 5.073 [4.517, 5.629] |
| 49 | 15 | Avg. screen-off bout [min.] | Adolescent |  | Week 1 | 38.55 [22.92, 54.17] |
| 50 | 15 | Avg. screen-off bout [min.] | Adolescent |  | Weeks >=5 | 24.68 [18.74, 30.63] |
| 51 | 15 | Avg. screen-off bout [min.] | Adult |  | Week 1 | 24.86 [14.14, 35.59] |
| 52 | 15 | Avg. screen-off bout [min.] | Adult |  | Weeks >=5 | 27.36 [22.52, 32.21] |
| 53 | 16 | Log(screen-on bout count) | Adolescent |  | Week 1 | 4.115 [3.889, 4.341] |
| 54 | 16 | Log(screen-on bout count) | Adolescent |  | Weeks >=5 | 4.203 [4.050, 4.356] |
| 55 | 16 | Log(screen-on bout count) | Adult |  | Week 1 | 4.114 [3.951, 4.278] |
| 56 | 16 | Log(screen-on bout count) | Adult |  | Weeks >=5 | 4.080 [3.956, 4.204] |

Model no. -- linear mixed-effects model (LMM) fit index; CI -- confidence interval; avg. -- average; min. -- minutes; log() - natural logarithm transformation.

**Table S3**. Estimated daily measure contrast (difference between strata) means across different sample strata by age group (adolescent vs. adult). Contrast means were estimated for the following daily screen time measures: total screen-on time (minutes), average screen-on bout duration (minutes), average screen-off bout duration (minutes), screen-on bout count (natural logarithm-transformed). The model fit number ("Model fit no.") represents the index of an individual linear mixed-effects model fit used to estimate contrast mean and 95% confidence interval of the mean in a given row.

| Row no. | Model fit no. | Daily measure (outcome) | Contrast (difference between strata) | Age group (covariate) | Contrast mean [95% CI] (p-value) |
| --- | --- | --- | --- | --- | --- |
| 1 | 1 | Total screen-on time [min.] | Adult - Adolescent |  | 16.45 [-13.38, 46.28] (0.280) |
| 2 | 2 | Avg. screen-on bout [min.] | Adult - Adolescent |  | 0.764 [-0.097, 1.626] (0.082) |
| 3 | 3 | Avg. screen-off bout [min.] | Adult - Adolescent |  | 1.003 [-6.482, 8.488] (0.793) |
| 4 | 4 | Log(screen-on bout count) | Adult - Adolescent |  | -0.101 [-0.296, 0.093] (0.307) |
| 5 | 5 | Total screen-on time [min.] | Android - iOS | Adolescent | -5.913 [-69.52, 57.70] (0.855) |
| 6 | 5 | Total screen-on time [min.] | Android - iOS | Adult | 5.854 [-32.39, 44.10] (0.764) |
| 7 | 6 | Avg. screen-on bout [min.] | Android - iOS | Adolescent | -0.157 [-1.995, 1.681] (0.867) |
| 8 | 6 | Avg. screen-on bout [min.] | Android - iOS | Adult | 0.141 [-0.963, 1.246] (0.802) |
| 9 | 7 | Avg. screen-off bout [min.] | Android - iOS | Adolescent | -6.368 [-22.29, 9.555] (0.433) |
| 10 | 7 | Avg. screen-off bout [min.] | Android - iOS | Adult | 0.827 [-8.752, 10.41] (0.866) |
| 11 | 8 | Log(screen-on bout count) | Android - iOS | Adolescent | 0.107 [-0.308, 0.521] (0.614) |
| 12 | 8 | Log(screen-on bout count) | Android - iOS | Adult | -0.070 [-0.318, 0.179] (0.583) |
| 13 | 9 | Total screen-on time [min.] | Weeks >=5 - (Weeks 1-4) | Adolescent | 11.40 [-4.430, 27.23] (0.158) |
| 14 | 9 | Total screen-on time [min.] | Weeks >=5 - (Weeks 1-4) | Adult | 6.944 [-5.391, 19.28] (0.270) |
| 15 | 10 | Avg. screen-on bout [min.] | Weeks >=5 - (Weeks 1-4) | Adolescent | 0.201 [-0.193, 0.595] (0.318) |
| 16 | 10 | Avg. screen-on bout [min.] | Weeks >=5 - (Weeks 1-4) | Adult | 0.372 [0.064, 0.679] (0.018) |
| 17 | 11 | Avg. screen-off bout [min.] | Weeks >=5 - (Weeks 1-4) | Adolescent | -6.122 [-12.21, -0.029] (0.049) |
| 18 | 11 | Avg. screen-off bout [min.] | Weeks >=5 - (Weeks 1-4) | Adult | 1.648 [-3.103, 6.398] (0.497) |
| 19 | 12 | Log(screen-on bout count) | Weeks >=5 - (Weeks 1-4) | Adolescent | 0.019 [-0.084, 0.122] (0.711) |
| 20 | 12 | Log(screen-on bout count) | Weeks >=5 - (Weeks 1-4) | Adult | -0.041 [-0.121, 0.040] (0.319) |
| 21 | 13 | Total screen-on time [min.] | Weeks >=5 - Week 1 | Adolescent | -1.069 [-26.12, 23.99] (0.933) |
| 22 | 13 | Total screen-on time [min.] | Weeks >=5 - Week 1 | Adult | 14.12 [-2.915, 31.16] (0.104) |
| 23 | 14 | Avg. screen-on bout [min.] | Weeks >=5 - Week 1 | Adolescent | -0.140 [-0.858, 0.579] (0.703) |
| 24 | 14 | Avg. screen-on bout [min.] | Weeks >=5 - Week 1 | Adult | 0.431 [-0.056, 0.919] (0.083) |
| 25 | 15 | Avg. screen-off bout [min.] | Weeks >=5 - Week 1 | Adolescent | -13.86 [-29.30, 1.572] (0.078) |
| 26 | 15 | Avg. screen-off bout [min.] | Weeks >=5 - Week 1 | Adult | 2.501 [-8.054, 13.06] (0.642) |
| 27 | 16 | Log(screen-on bout count) | Weeks >=5 - Week 1 | Adolescent | 0.088 [-0.100, 0.275] (0.359) |
| 28 | 16 | Log(screen-on bout count) | Weeks >=5 - Week 1 | Adult | -0.035 [-0.162, 0.093] (0.596) |

Model no. -- linear mixed-effects model (LMM) fit index; CI -- confidence interval; avg. -- average; min. -- minutes; log() - natural logarithm transformation.

**
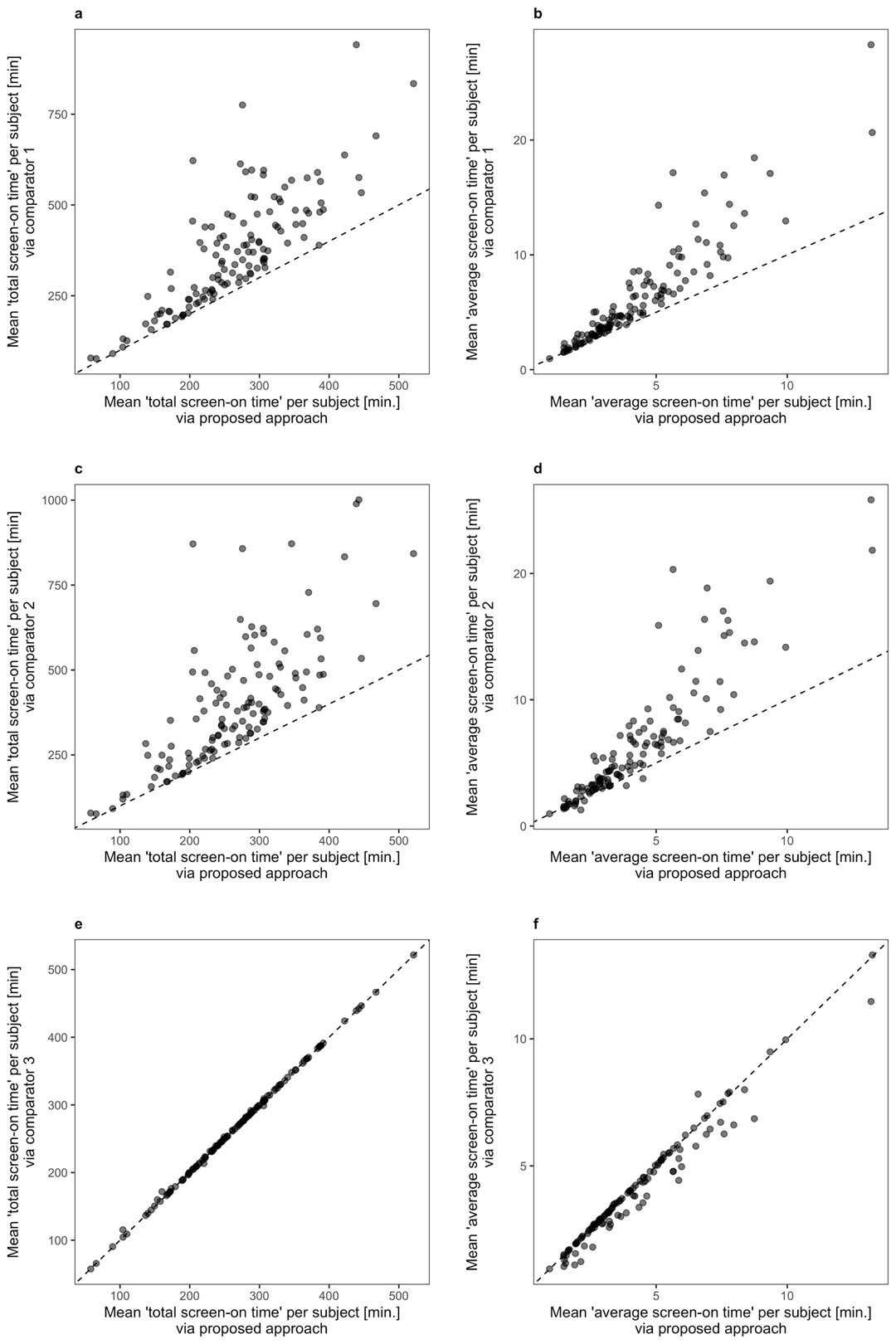
**

**Figure S1**. Comparison of mean total screen-on time (plots **a, c, d**) and screen-on time bout duration (plot **b, d, e**) when calculated via the proposed approach (x-axis) and comparator approaches: (1) imputing missing logs and capping screen-on bout duration at 6 hours (instead of 30 minutes) (plots **a, b**); (2) not imputing missing logs i.e. only considering consecutive pairs of matching events ("unlocked" and "locked" for iOS, "screen turned on" and "screen turned off" for Android) and not capping screen-on bout duration (i.e., minimal preprocessing) (plots **c, d**); (3) not imputing missing logs with screen-on bout duration capped at 30 minutes (plots **e, f**). Each point represents a mean of daily measure calculated for one participant across their monitoring days. Dashed line is a 45-degree line.

**
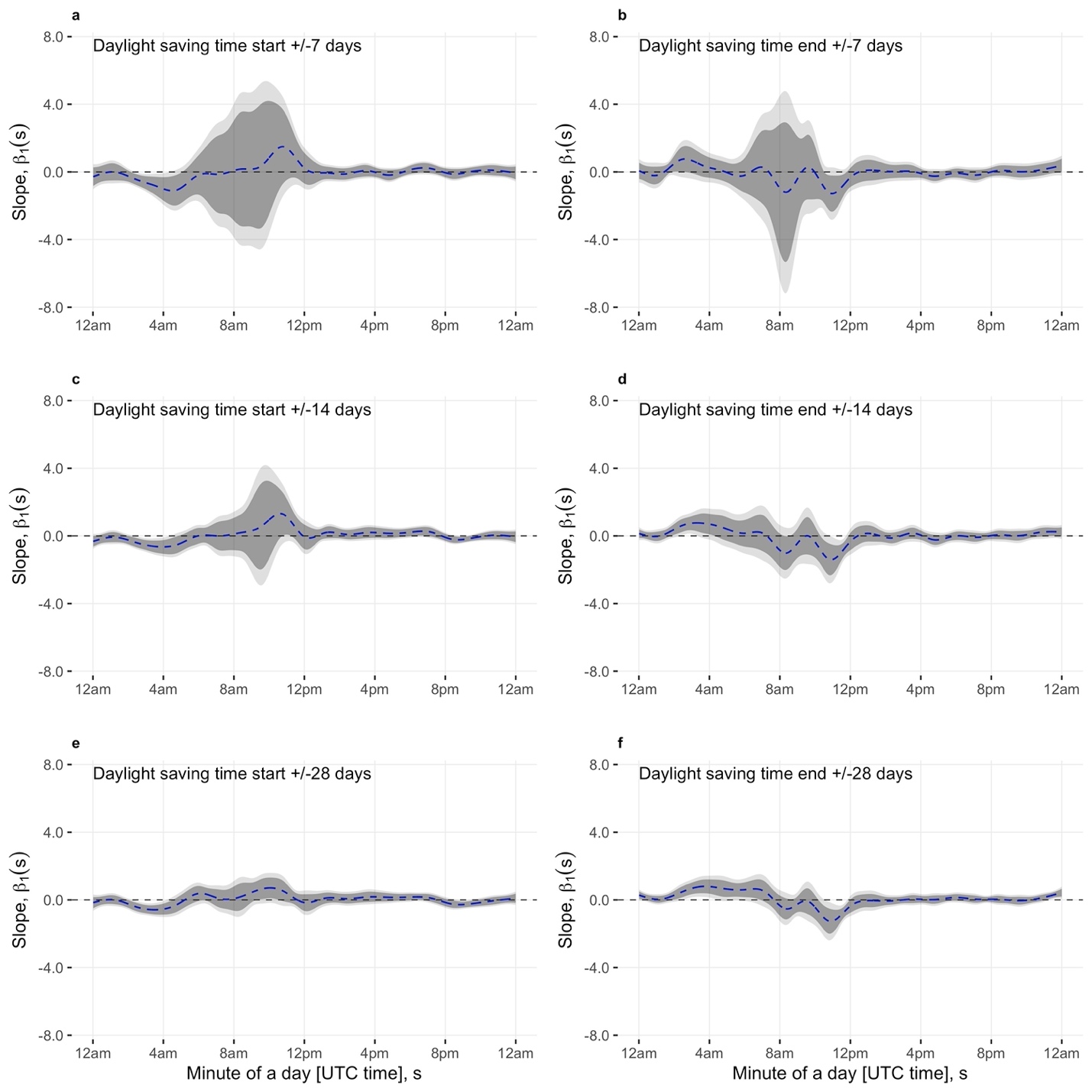
**

**Figure S2**. Effect of daylight saving time change on probability of a screen time in a given minute across minute-level functional domain of a day. The logistic function-on-scalar mixed-effects regression model estimates the effect of the covariate (1=after, 0=before daylight saving time change) on the functional outcome (1=any screen time, 0=no screen time) across minutes of the day (y-axis). The solid black line represents the functional coefficient estimates, while the dark gray shaded area represents the 95% point-wise confidence intervals, and the light gray shaded area represents the 95% joint confidence intervals. The plots are divided into three sets for data from daylight saving time start (plots **a, c, e**) and end (plots **b, d, f**), with data from different window sizes used in the modeling: +/- 7 (plots **a, b**), +/- 14 (plots **c, d**) +/- 28 (plots **e, f**).

# METHODS

## Collection of phone state logs

## We collected phone state logs using the Beiwe smartphone application, which is the frontend of the Beiwe open-source high-throughput digital phenotyping platform. Participants were assigned a Beiwe participant ID and had the Beiwe app installed during their inpatient unit stay (adolescents; phones were not allowed in the unit, so the guardian of a patient would temporarily bring the device to the unit to have the app installed) or during their emergency room visit (adults). Participants were followed up for up to 6 months after discharge, where the day of discharge defined the study start date. For some adolescent participants who were discharged to another healthcare facility where phone use was not allowed, we estimated the date of their return home based on the program they attended. Phone state logs collected before their return home were not used in analyses (see "Missing data labeling" subsection in the main text).

## The phone state logs recorded events with millisecond timestamps. The type of information available in the state logs differs by the smartphone OS. **Table S1** lists the possible events in the state logs. For Android, phone state events include "screen turned on" and "screen turned off", while for iOS, phone state events include "locked" and "unlocked". In addition, each iOS phone state event includes the current battery level, with each change of the battery level by 1% (increase or decrease) recorded in the log.

## Estimating the timing and duration of screen-on bouts at a subsecond level

We defined a smartphone "screen-on bout" as a period of consecutive time spent using a smartphone, and a "screen-off bout" as a period of consecutive time spent not using a smartphone, such that a screen-on bout is always followed by a screen-off bout, and vice versa.

We used phone state logs to estimate the timing and duration of screen-on bouts at a subsecond level. For iOS, the estimation was based on the time intervals between subsequent "unlocked" and "locked" event timestamps in the log. We took a few preprocessing steps to prepare the data for analysis.

First, we found that 1.1% of "locked" events were followed by another "locked" event (and not "unlocked" as expected), and similarly, 1.01% of "unlocked" events were followed by another "unlocked" event (and not "locked").

- For two consecutive "locked" events, to impute the missing events in the log, we inserted an "unlocked" event 60 seconds before the second of two consecutive "locked" events, provided the time between them was more than 60 seconds. If the time between them was at most 60 seconds, we inserted the "unlocked" event in the middle of the two consecutive "locked" events.
- For two consecutive "unlocked" events, to impute the missing events in the log, we inserted a "locked" event 60 seconds after the first of the two consecutive "unlocked" events, provided the time between them was more than 60 seconds. If the time between them was at most 60 seconds, we inserted the "locked" event in the middle of the two consecutive "unlocked" events.

Second, we winsorized screen-on bouts longer than 30 minutes (3.37%) by updating their end time, so that the bout duration was 30 minutes. Winsorizing is a statistical technique that replaces extreme values in a dataset with less extreme values to reduce the impact of outliers on statistical analysis."

For Android, the estimation was based on the time intervals between subsequent "screen turned on" and "screen turned off" event timestamps. First, we performed similar "missing" event imputation step to address the case that 0.17% of "screen turned off" events were followed by another "screen turned off" event (and not "screen turned on" as expected), and 0.76% of "screen turned on" events were followed by another "screen turned on" event (and not "screen turned off").

- For two consecutive "screen turned off" events, if the time between them was more than 60 seconds, the "screen turned on" event was inserted 60 seconds before the second of the two consecutive "screen turned off" events. If the time between them was at most 60 seconds, the "screen turned on" event was inserted in the middle of the two consecutive "screen turned off" events.
- For two consecutive "screen turned on" events, if the time between them was more than 60 seconds, the "screen turned off" event was inserted 60 seconds after the first of the two consecutive "screen turned on" events. If the time between them was at most 60 seconds, the "screen turned off" event was inserted in the middle of the two consecutive "screen turned on" events.

Second, we also winsorized screen-on bouts longer than 30 minutes (3.41%). Third, we identified the bouts which likely were the result of a screen turned on due to a smartphone notification rather then intentional phone use. This was done by identifying and removing short (no longer than 40 seconds) screen-on bouts of the same duration (expressed in seconds, after rounding to 1 decimal place) that occurred repetitively. The "repetitive" bout was defined as a bout that occurs at least 3 times and accounts for more than 1% of all bouts in a given week. The screen-on bouts removed in this step accounted for 15.79% of bouts.

Our preprocessing steps closely followed those presented in Kristensen and colleagues. Compared to their approach, we used slightly different parameter values for the winsorizing and notification removal algorithm:

- In both Kristensen and colleagues and our work, for both iOS and Android, for missing event imputation, an event was imputed yielding an screen-on bout of 60 seconds. We also specify the case when the time between the two events (with a missing intermediate event) is less than 60 seconds (see above).
- In Kristensen and colleagues, for both iOS and Android, the winsorizing of screen-on bout duration was done at 6 hours. In our work, for both iOS and Android, the winsorizing of screen-on bout duration was done at 30 minutes.
- In Kristensen and colleagues, for Android, the notification removal algorithm was run on day-level data for each individual, removing bouts accounting for at least 1% of the events and recurring at least 3 times. In our work, for Android, the notification removal algorithm was run on week-level data for each individual, removing bouts accounting for at least 1% of the events and recurring at least 3 times during a week-long period.
